# Risk of flare or relapse in patients with immune-mediated diseases following SARS-CoV-2 vaccination: a systematic review and meta-analysis

**DOI:** 10.1186/s40001-024-01639-4

**Published:** 2024-01-17

**Authors:** Mahya Shabani, Parnian Shobeiri, Shadi Nouri, Zahra Moradi, Robel Assefa Amenu, Mohammad-Mehdi Mehrabi Nejad, Nima Rezaei

**Affiliations:** 1 School of Medicine, Tehran University of Medical Sciences, Tehran, Iran; 2 Non-Communicable Diseases Research Center, Endocrinology and Metabolism Population Sciences Institute, Tehran University of Medical Sciences, Tehran, Iran; 3 Department of Immunology, Research Center for Immunodeficiencies, Pediatrics Center of Excellence, Children’s Medical Center, Tehran University of Medical Sciences, Qarib St, Keshavarz Blvd, 14194, Tehran, 1419733141 Iran; 4 Network of Immunity in Infection, Malignancy and Autoimmunity (NIIMA), Universal Scientific Education and Research Network (USERN), Tehran, Iran; 5 Arak University of Medical Sciences, Arak, Iran; 6 Institute of Public Health and Clinical Nutrition, University of Eastern Finland, Kuopio, Finland; 7 Department of Radiology, School of Medicine, Advanced Diagnostic and Interventional Radiology Research Center (ADIR), Imam Khomeini Hospital, Tehran University of Medical Sciences (TUMS), Qarib St, Keshavarz Blvd, 14194, Tehran, 1419733141 Iran

**Keywords:** Autoimmune disease, mRNA, Vector-based vaccine, COVID-19, Vaccine

## Abstract

**Background:**

Patients with autoimmune and immune-mediated diseases (AI-IMD) are at greater risk of COVID-19 infection; therefore, they should be prioritized in vaccination programs. However, there are concerns regarding the safety of COVID-19 vaccines in terms of disease relapse, flare, or exacerbation. In this study, we aimed to provide a more precise and reliable vision using systematic review and meta-analysis.

**Methods:**

PubMed-MEDLINE, Embase, and Web of Science were searched for original articles reporting the relapse/flare in adult patients with AI-IMD between June 1, 2020 and September 25, 2022. Subgroup analysis and sensitivity analysis were conducted to investigate the sources of heterogeneity. Statistical analysis was performed using R software.

**Results:**

A total of 134 observations of various AI-IMDs across 74 studies assessed the rate of relapse, flare, or exacerbation in AI-IMD patients. Accordingly, the crude overall prevalence of relapse, flare, or exacerbation was 6.28% (95% CI [4.78%; 7.95%], *I*^2^ = 97.6%), changing from 6.28% (*I*^2^ = 97.6%) to 6.24% (*I*^2^ = 65.1%) after removing the outliers. AI-IMD patients administering mRNA, vector-based, and inactive vaccines showed 8.13% ([5.6%; 11.03%], *I*^2^ = 98.1%), 0.32% ([0.0%; 4.03%], *I*^2^ = 93.5%), and 3.07% ([1.09%; 5.9%], *I*^2^ = 96.2%) relapse, flare, or exacerbation, respectively (*p*-value = 0.0086). In terms of disease category, nephrologic (26.66%) and hematologic (14.12%) disorders had the highest and dermatologic (4.81%) and neurologic (2.62%) disorders exhibited to have the lowest crude prevalence of relapse, flare, or exacerbation (*p*-value < 0.0001).

**Conclusion:**

The risk of flare/relapse/exacerbation in AI-IMD patients is found to be minimal, especially with vector-based vaccines. Vaccination against COVID-19 is recommended in this population.

**Supplementary Information:**

The online version contains supplementary material available at 10.1186/s40001-024-01639-4.

## Introduction

Among the general population, patients with autoimmune and immune-mediated diseases (AI-IMD) are at greater risk of COVID-19 infection due to their underlying disease-related immune dysfunction along with the immunosuppressive treatments [1]. Increased morbidity, mortality, and costs are attributed to AI-IMD flares [2] highlighting the significance of disease activity control during this pandemic. There is also evidence supporting disease relapse after COVID-19 in MS patients [3].

Vaccination is considered the best strategy to effectively reduce COVID-19-related morbidity and mortality [4]. Approved vaccines against SARS-CoV-2 are categorized into different main types including mRNA, vector-based, and inactive [5]. Concern regarding the vaccine’s suboptimal efficacy and safety, especially vaccine-induced flare, is shown to have the strongest association with vaccine hesitancy among AI-IMD patients [6]. Although vaccines are generally safe, several studies reported SLE flare following influenza and papilloma vaccines [7–9].

There are different technologies for developing SARS-CoV-2 vaccines, including inactivated and nucleic-acid vaccines composed of mRNA or plasmid or viral DNA vectors, which code for a specific antigen. To achieve a robust long-lasting immunogenicity in both humoral and cellular immune systems, an adjuvant component is added to the antigen activating three pathways [10]. Major histocompatibility complex–T cell receptor (MHC–TCR) interaction (specific), costimulatory signal to TCR (non-specific), and pro-inflammatory signals (non-specific) using cytokines to develop Th1, Th2, and Th17 from T lymphocytes [11]. Adjuvants also trigger innate immunity through toll-like receptors (TLRs) [12]. Although these components are critical for robust immunity, they might also initiate an undesired immune response and trigger autoimmune disease relapse [13]. Besides, the abundance of cytokines produced during this process can result in the reactivation of reminiscent self-reacting lymphocyte clones through bystander activation and blunt the mechanisms of tolerance [14].

Data on SARS-CoV-2 vaccine safety in this vulnerable population are limited as they were widely excluded from the original vaccine trials; however, it is increasingly investigated through different clinical trials [15, 16]. Despite the ample evidence in the literature investigating the immunogenicity of COVID-19 vaccines in AI-IMD patients, their safety profile, particularly disease flare/relapse, has been less studied [5, 17]. There is inconsistency regarding the safety of COVID-19 vaccines in AI-IMD patients; hence, we aimed to provide a more precise and reliable vision using systematic review and meta-analysis.

## Materials and methods

### Protocol and literature search

This systematic review and meta-analysis study was carried out according to the Preferred Reporting Items for Systematic Reviews and Meta-Analyses (PRISMA) guidelines.

PubMed-MEDLINE, Embase, and Web of Science were searched for original articles reporting the relapse/flare in adult patients with AI-IMD between June 1, 2020, and October 1, 2022. The search terms were as follows: ((COVID) OR (COVID-19) OR (SARS-CoV-2) OR (novel coronavirus)) AND ((vaccine) OR (vaccination)) OR (vaccinated)) AND ((Flare) OR (relapse) OR (Flare-up) OR (exacerbation) OR (recurrence)) AND ((autoimmune) OR (rheumatology) OR (rheumatologic disease) OR (Rheumatoid arthritis) OR (RA) OR (Systemic lupus erythematosus) OR (SLE) OR (Guillain–Barre syndrome) OR (Multiple sclerosis) OR (Myasthenia gravis) OR (Psoriasis) OR (Inflammatory bowel disease) OR (Graves' disease) OR (Sjögren's syndrome) OR (Hashimoto's thyroiditis) OR (vasculitis) OR (Crohn's disease) OR (ulcerative colitis) OR (Nephropathy) OR (Pemphigus Vulgaris) OR (bullous pemphigoid) OR (Immune thrombocytopenia) OR (dermatomyositis) OR (polymyositis)).

Two reviewers independently conducted the literature search, and any disagreement was resolved by discussion or consultation with a third expert. The authors were not blinded to the data of the articles, including the author, institution, or journal, while screening studies or extracting data. EndNote version × 20 was used for literature management.

### Eligibility criteria

Studies exploring the prevalence of disease relapse/flare/exacerbation following COVID-19 vaccination in AI-IMD patients were eligible for inclusion. The included studies met the following criteria: (1) population: studies on AI-IMD patients. AI-IMD patients included patients with (a) rheumatic and musculoskeletal diseases (including rheumatoid arthritis, SLE, vasculitis, ankylosing spondylitis, dermatomyositis, polymyositis, Systemic sclerosis, Behcet syndrome, etc.); (b) neurologic diseases (including MS, myasthenia gravis, Guillain–Barré syndrome, demyelinating polyneuropathy, etc.); (c) gastroenterologic diseases (including Crohn's disease, ulcerative colitis, etc.); (d) dermatologic diseases (including Pemphigus Vulgaris, Bullous Pemphigoid, Psoriasis, etc.); (e) hematologic diseases (including immune thrombocytopenic purpura (ITP); mixed cryoglobulinaemic vasculitis, etc.); and (f) nephrologic diseases (including nephrotic syndrome). (2) Intervention: COVID-19 vaccination. (3) Study design: all cross-sectional, observational, retrospective, and prospective studies were included. (4) Outcomes: the primary outcome of this study was disease relapse/flare/exacerbation following COVID-19 vaccination in AI-IMD patients after COVID-19 vaccination. The exclusion criteria were as follows: (1) case reports or case series patients; (2) non-original studies including reviews and editorials; (3) partially overlapping patient cohorts; (4) not reporting the relapse/flare percentage after COVID-19 vaccination; (5) articles not written in English; and (6) non-human studies. Two reviewers independently screened the literature in consensus.

### Data extraction

Two groups of reviewers independently evaluated eligible studies and recorded the following data: the first author, publication year, country of origin, study design, studied disease, inclusion and exclusion criteria, study sample size, the number of AI-IMD patients, female percentage, mean (SD)/median [IQR] of age, flare or relapse or exacerbation and its percentage, and the type of vaccine. Any disagreement in data extraction was resolved by consensus or consultation with a third expert.

### Quality assessment

The National Institutes of Health (NIH) quality assessment tool [18] was employed to assess the quality of the included studies. The scores of 11–14, 6–10, and 0–5 were considered good, fair, and poor quality, respectively. Furthermore, two independent expert reviewers assessed the included studies in terms of methodology; any conflict was resolved by consensus.

### Statistical analysis

We used the 'metaprop' function and the Der Simonian and Laird random-effect model to assess the pooled effect of the prevalence of relapse, flare, or exacerbation in AI-IMD patients. A forest plot was created to depict the summary of meta-analysis findings and heterogeneity. The funnel plot and Egger's regression tests were used to screen for publication bias, with a *p*-value of < 0.05 regarded to suggest probable publication bias. Cochrane's Q statistic was used to assess between-study heterogeneity. *I*^2^ was used to assess between-study heterogeneity, with values of 0 representing no heterogeneity, and 25, 50, and 75% representing low, medium, and increasing heterogeneity, respectively. All computations and visualizations were carried out using R version 4.2.1 (R Core Team [2020]. R: A language and environment for statistical computing. R Foundation for Statistical Computing, Vienna, Austria). We used the following packages: “meta” (version 4.17–0), “metafor” (version 2.4–0), “dmetar” (version 0.0–9), and “tidyverse” (version 1.3.0). All forest and funnel plots were designed using R. A *p*-value of < 0.05 was considered statistically significant.

## Results

### Overall prevalence of relapse/flare/exacerbation in AI-IMD patients AI-IMD

**Fig. 1:**
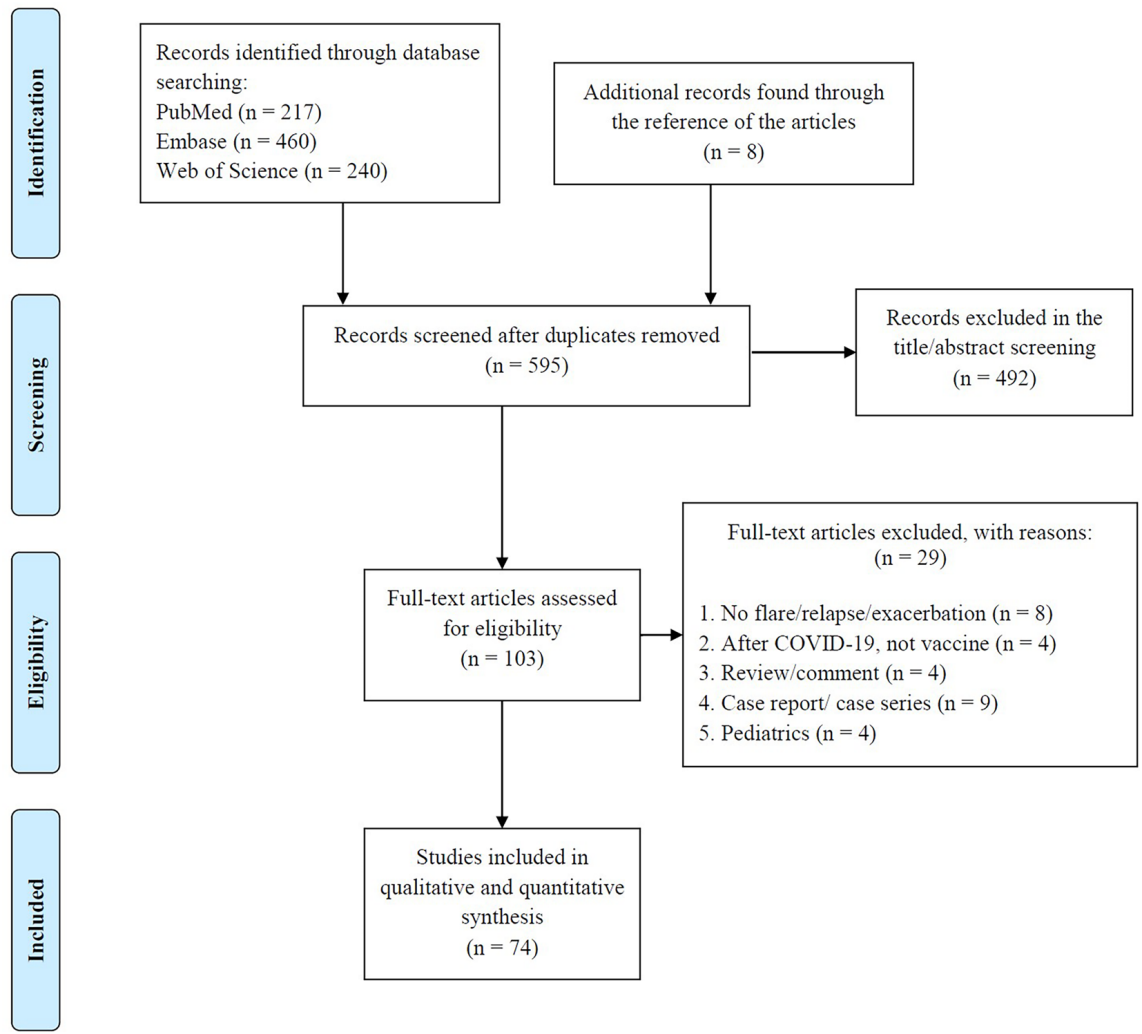
Study selection process according to the preferred reporting items for systematic reviews and meta-analyses (PRISMA) guideline. After evaluating the total of 595 studies, 74 studies met the eligibility criteria and used in qualitative and quantitative analyses

**Fig. 2:**
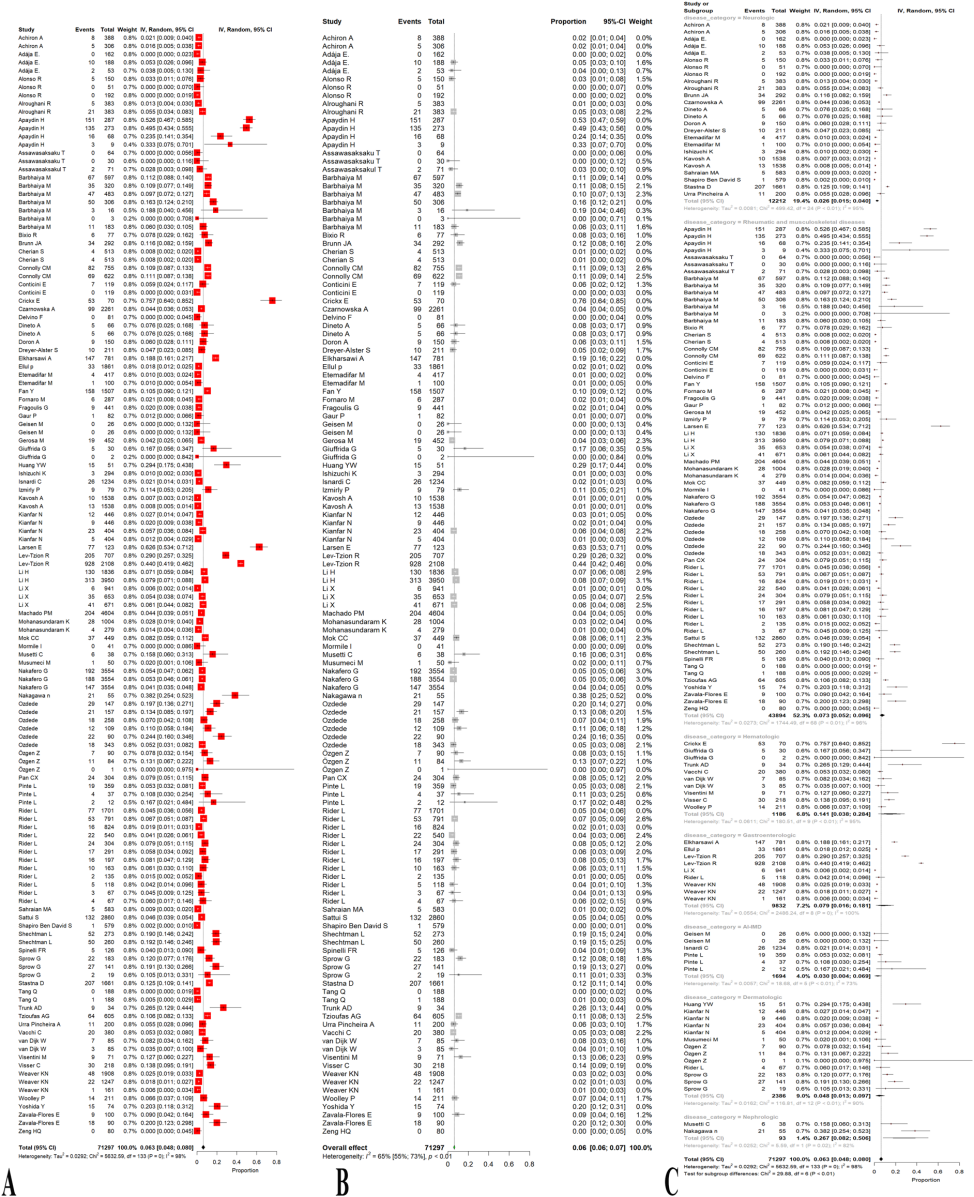
Forest plots representing the prevalence of relapse, flare, or exacerbation in all patients with autoimmune and immune-mediated diseases (AI-IMD) before (**A**) and after (**B**) removing the outliers and based on the type of AI-IMD disease (**C**) following the COVID-19 vaccination. The prevalence of relapse, flare, or exacerbation was statistically significantly different across the six disease categories overall, as shown by a *p*-value of < 0.0001

The study selection flowchart is presented in Fig. 1. A total of 134 observations of various AI-IMDs across 74 studies [19–92] assessed the rate of relapse, flare, or exacerbation in AI-IMD patients (Table 1). Accordingly, the overall crude prevalence of relapse, flare, or exacerbation was 6.28% (95% CI 4.78%; 7.95%, test of heterogeneity: *I*^2^ = 97.6%, *p*-value = 0, Fig. 2a).

**Table 1:** Details of the data presented by the included studies

First author	Year	Country	Study design	Disease category	Vaccine category	Total sample size	Female%	Age	
								Mean (SD)	Median [IQR]
Achiron A	2021	Israel	Observational	Neurologic (MS)	mRNA	555	65.6		
Adája E. Baars	2022	Netherlands	Prospective Cohort	Neurologic	mRNA and vector-based	403			
Alonso R	2021	Argentina	Cross-sectional	Neurologic (MS)	All	393	82.4	41.5 (11.8)	
Alroughani R	2022	Kuwait	Cross-sectional	Neurologic (MS)	mRNA and vector-based	647			
Apaydin H	2022	Turkey	Retrospective Cohort	Rheumatic and musculoskeletal diseases (Behcet syndrome)	mRNA and inactive	287	45.3		42 [34, 50]
Assawasaksaku T	2022	Thailand	Prospective Cohort	Rheumatic and musculoskeletal diseases (SLE)	All	94			
Assawasaksakul T	2022	Thailand	Prospective Cohort	Rheumatic and musculoskeletal diseases (SLE)	mRNA	71	95.8	39 (11.9)	
Barbhaiya M	2021	USA	Cross-sectional	Rheumatic and musculoskeletal diseases	mRNA/vector-based	1101	80.6	60.8 (14.2)	
Barbhaiya M	2021	USA	Retrospective Cohort	Rheumatic and musculoskeletal diseases (SLE)	mRNA and vector-based	183	94	52.5 (14.2)	
Bixio R	2021	Italy	Prospective Cohort	Rheumatic and musculoskeletal diseases	mRNA	77	80.5	62.2 (13.2)	
Brunn JA	2022	USA	Prospective Cohort	Neurologic (MS)	All	292	81.4	50.4 (12.4)	
Cherian S	2021	Germany	Cross-sectional	Rheumatic and musculoskeletal diseases	mRNA	513	82.65	58.46 (10.28)	
Connolly CM	2022	USA	Prospective Cohort	Rheumatic and musculoskeletal diseases	mRNA	1377	92		47 [37, 59]
Conticini E	2022	Italy	Prospective Cohort	Rheumatic and musculoskeletal diseases (idiopathic inflammatory myopathies)	mRNA and vector-based	119	73.1		58 [47, 66]
Crickx E	2021	UK	Prospective Cohort	Hematologic (ITP)	mRNA and vector-based	92	59.8		69 [24, 90]
Czarnowska A	2022	Poland	Cross-sectional	Neurologic (MS)	mRNA and vector-based	2261	70.5	42.6	
Delvino F	2021	Italy	Prospective Cohort	Rheumatic and musculoskeletal diseases (Giant cell arteritis)	mRNA	81	67.9	75.8 (6.9)	
Dinoto A	2021	Italy	Prospective Cohort	Neurologic (MS)	mRNA	66			
Doron A	2022	Israel	Retrospective Cohort	Neurologic (myasthenia gravis)	mRNA	160	44.4	57.2 (18)	
Dreyer-Alster S	2022	Israel	Prospective Cohort	Neurologic (myasthenia gravis)	mRNA	211	62		
Elkharsawi A	2022	Germany	Cross-sectional	Gastroenterologic	All	914	64.3		44 [34, 56]
Ellul p	2022	36 European countries	Cross-sectional	Gastroenterologic	All	3272	60.4		43 [33, 54]
Etemadifar M	2022	Iran	Retrospective Cohort	Neurologic (MS)	Inactive	517	76.79	37.81 (8.74)	
Fan Y	2021	China	Cross-sectional	Rheumatic and musculoskeletal diseases	Inactive	1507	77.4		39 [31, 51]
Fornaro M	2022	Italy	Prospective Cohort	Rheumatic and musculoskeletal diseases	mRNA	452	83.3	53 (4)	
Fragoulis G	2022	Greece	Cross-sectional	Rheumatic and musculoskeletal diseases	All	561	75.6	54.4 (14.8)	
Gaur P	2021	India	Cross-sectional	Rheumatic and musculoskeletal diseases	Vector-based	280	83.3	47 (13)	
Geisen M	2021	Germany	Prospective Cohort	AI-IMD	mRNA	26	64.3	50.5 (15.8)	
Gerosa M	2022	Italy	Retrospective Cohort	Rheumatic and musculoskeletal diseases (SLE)	mRNA and vector-based	452	92.25		48 [35, 56]
Giuffrida G	2022	Italy	Prospective Cohort	Hematologic (ITP)	mRNA	32			47 [19, 73]
Huang YW	2021	Taiwan	Prospective Cohort	Dermatologic (Psoriasis)	mRNA and vector-based	83			
Ishizuchi K	2022	Japan	Prospective Cohort	Neurologic (myasthenia gravis)	mRNA and vector-based	343	65.3	57	
Isnardi C	2022	Argentina	Retrospective Cohort	AI-IMD	All	1234	79	57.8 (14.1)	
Izmirly P	2022	USA	Prospective Cohort	Rheumatic and musculoskeletal diseases (SLE)	mRNA and vector-based	90	87.8	45.5 (14.2)	
Kavosh A	2022	Iran	Cross-sectional	Neurologic (MS)	Inactive	1538	74.8	40.45 (9.74)	
Kianfar N	2022	Iran	Cross-sectional	Dermatologic	Vector-based and inactive	446	54.7	50.2 (12.5)	
Larsen E	2022	Denmark	Prospective Cohort	Rheumatic and musculoskeletal diseases (SLE)	mRNA and vector-based	123	89.4		51 [42, 63]
Lev-Tzion R	2022	Israel	Cross-sectional	Gastroenterologic	mRNA	4946	51	51 (16)	
Li H	2022	UK	case-crossover	Rheumatic and musculoskeletal diseases (Gout)	mRNA/vector-based	5904	14.5	63.1 (14.7)	
Li X	2021	China	Cross-sectional	Gastroenterologic	mRNA	941			
Li X	2021	China	Retrospective Cohort	Rheumatic and musculoskeletal diseases (RA)	mRNA/inactive	5493			
Machado PM	2022	UK (data from 30 countries)	Cross-sectional	Rheumatic and musculoskeletal diseases	mRNA and vector-based	5121	70	61.6 (15.2)	
Mohanasundaram K	2022	India	Cross-sectional	Rheumatic and musculoskeletal diseases	Vector-based/inactive	2092	78.7	47.5 (13.17)	
Mok CC	2022	Hong Kong	Retrospective Cohort	Rheumatic and musculoskeletal diseases (SLE)	mRNA and inactive	914	92.5	48.6 (14.0)	
Mormile I	2022	Italy	Prospective Cohort	Rheumatic and musculoskeletal diseases (SLE)	mRNA	41	87	26 (11)	
Musetti C	2022	Italy	Retrospective Cohort	Nephrologic	mRNA and vector-based	38	26.3	45.9 (19.1)	
Musumeci M	2021	Italy	Prospective Cohort	Dermatologic (Psoriasis)	mRNA	50	44		Range: 33–83 years)
Nakafero G	2022	UK	Cross-sectional	Rheumatic and musculoskeletal diseases	mRNA and vector-based	3554	71.8	65 (15)	
Nakagawa n	2022	Japan	Cross-sectional	Nephrologic	mRNA	55	44.4		
Ozdede	2022	Turkey	Cross-sectional	Rheumatic and musculoskeletal diseases	mRNA/inactive	256	37.9	43.21 (10.13)	
Özgen Z	2022	Turkey	Cross-sectional	Dermatologic (pemphigus vulgaris)	Inactive/mRNA/vector-based	244	52.9		
Pan CX	2022	USA	Retrospective Cohort	Rheumatic and musculoskeletal diseases (dermatomyositis)	All	304	83.2		
Pinte L	2021	Romania	Prospective Cohort	AI-IMD	mRNA/vector-based	416	81.5		50 [21, 88]
Rider L	2022	USA	Retrospective Cohort	Rheumatic and musculoskeletal diseases/Gastroenterologic/Dermatologic	All	5619	85.7		55.5 [44.4,65.4]
Sahraian MA	2021	Iran	Cross-sectional	Neurologic (MS)	Inactive	583	78	36.2 (8.2)	
Sattui S	2021	USA	Cross-sectional	Rheumatic and musculoskeletal diseases	All	2860	86.7	55.3	
Shapiro Ben David S	2021	Israel	Retrospective Cohort	Neurologic (Guilain barre)	mRNA	702	48	53 (18)	
Shechtman L	2022	Israel	Cross-sectional	Rheumatic and musculoskeletal diseases	mRNA	273	54.5	41 (15.5)	
Spinelli FR	2022	Italy	observational	Rheumatic and musculoskeletal diseases	mRNA	126	83.3		51 [34, 68]
Sprow G	2022	USA	Retrospective Cohort	Dermatologic	mRNA/vector-based	402	81.6		58 [95%CI 56, 95%CI 60]
Stastna D	2022	Czech Republic	Retrospective Cohort	Neurologic (MS)	mRNA and vector-based	1661	72.37	48.49 (11.43)	
Tang Q	2022	China	Cross-sectional	Rheumatic and musculoskeletal diseases (SLE)	Inactive	378			
Trunk AD	2021	USA	Retrospective Cohort	Hematologic (Chronic graft-versus-host disease (CGVHD))	mRNA	34			
Tzioufas AG	2021	Greece	Prospective Cohort	Rheumatic and musculoskeletal diseases	mRNA	605	71.4		58 [range: 16–91] [,]
Urra Pincheira A	2022	Canada	Retrospective Cohort	Neurologic (myasthenia gravis)	mRNA and vector-based	200	48.5	64.3 (13.9)	
Vacchi C	2022	Italy	Cross-sectional	Hematologic (Mixed cryoglobulinaemic vasculitis (MCV))	All	416	68	70.42 (11.75)	
van Dijk W	2021	Netherlands	Retrospective Cohort	Hematologic (ITP)		85	53	48 (17)	
Visentini M	2022	Italy	Prospective Cohort	Hematologic	mRNA and vector-based	71			
Visser C	2021	Netherlands	observational	Hematologic (ITP)	mRNA and vector-based	418			
Weaver KN	2021	USA	Prospective Cohort	Gastroenterologic	mRNA and vector-based	3316	71.7	43.7 (15.1)	
Woolley P	2022	UK	Prospective Cohort	Hematologic (ITP)	mRNA and vector-based	294			
Yoshida Y	2022	Japan	Prospective Cohort	Rheumatic and musculoskeletal diseases (SLE)	mRNA	74	96	50 (14)	
Zavala-Flores E	2021	Peru	observational	Rheumatic and musculoskeletal diseases (SLE)	mRNA	100	94	38.9	
Zeng HQ	2022	China	Cross-sectional	Rheumatic and musculoskeletal diseases	Inactive	80	70	40.85 (9.50)

After removing the outliers [19–24, 26, 27, 31, 33–35, 40–46, 50–52, 54–58, 60, 62, 63, 65, 68–70, 74–77, 79–82, 87, 88, 90, 91], the prevalence of relapse, flare, or exacerbation was 6.24% (95% CI 5.57%; 6.95%, test of heterogeneity: *I*^2^ = 65.1%, *p*-value < 0.0001, Fig. 2b).

**Fig. 3:**
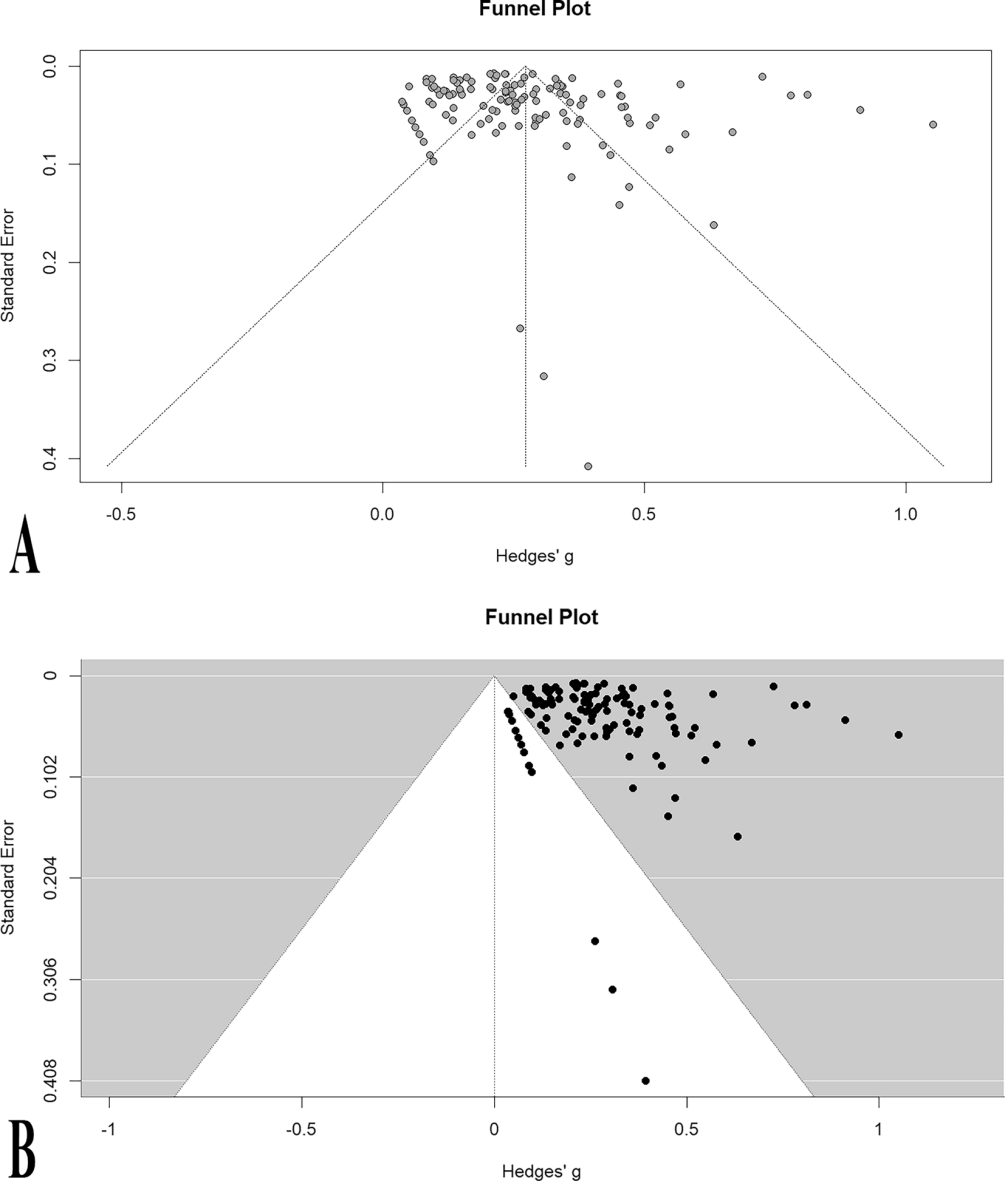
Funnel plots before (**A**) and after (**B**) removing the outliers representing no publication bias

Regarding the publication bias, Egger’s test did not corroborate funnel plot asymmetry as well as the illustrated funnel plot (*p*-value = 0.27, Fig. 3).

### Subgroup analysis

#### By vaccine category

**Fig. 4:**
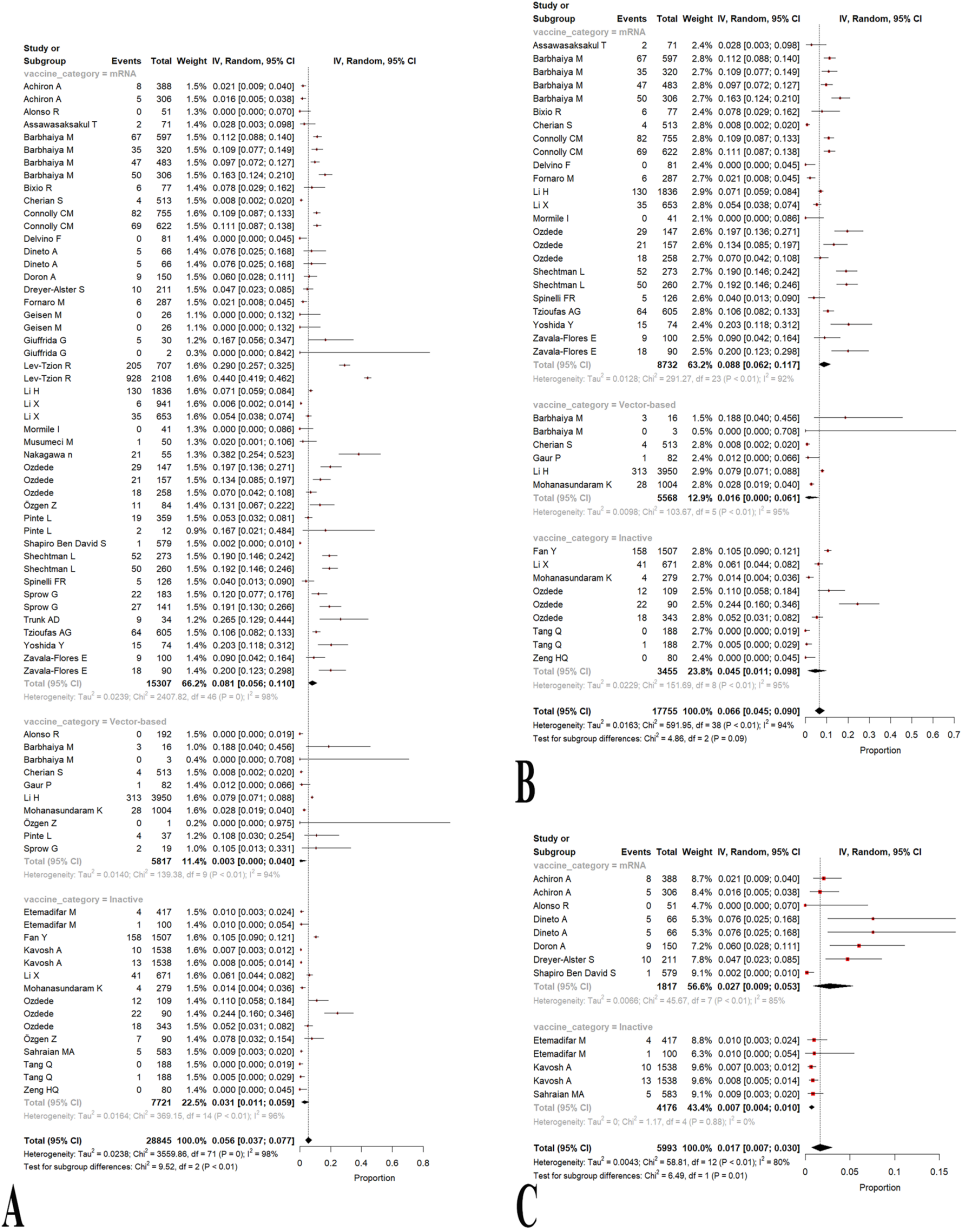
Forest plots representing the prevalence of relapse, flare, or exacerbation based on the type of vaccine in all patients with autoimmune and immune-mediated diseases (AI-IMD) (*p*-value = 0.0086) (**A**), patients with rheumatic and musculoskeletal diseases (*p*-value = 0.0882) (**B**), and neurologic (*p*-value = 0.0108) (**C**) autoimmune diseases following the COVID-19 vaccination

Considering the administered vaccine category as mRNA, vector-based, and inactive vaccines, we carried out a subgroup analysis consisting of 47, 10, and 15 observations, respectively. AI-IMD patients administering mRNA, vector-based, and inactive vaccines showed 8.13% (95% CI 5.6%; 11.03%, test of heterogeneity: I^2^ = 98.1%), 0.32% (95% CI 0.0%; 4.03%, test of heterogeneity: *I*^2^ = 93.5%), and 3.07% (95% CI 1.09%; 5.9%, test of heterogeneity: *I*^2^ = 96.2%) relapse, flare, or exacerbation, respectively (Fig. 4a; Table 2). Overall, a *p*-value of 0.0086 demonstrated a significant statistical difference in the prevalence of relapse, flare, or exacerbation between these three vaccine categories. Of note, some studies utilized a mixture of vaccine platforms, and accordingly, they were not eligible to enter as an observation in the proposed subgroup meta-analysis. Additionally, the results of the pair-wised analysis of the vaccine category are stated in Table 2, showing that only mRNA vs. inactivated vaccine platforms have a statistically significant difference in the prevalence of relapse, flare, or exacerbation.

**Table 2:** Results of between-group meta-analyses based on type of vaccine and disease category

Sub-group	Comparison	No. studies	No. participants	No. events	Meta-analysis			Heterogeneity	
					Effect size (%)	95% Confidence interval (%)	*p* value	*I*^2^ (%)	*p* value
Type of vaccine pair-wised	mRNA vs. inactivated	38	23,028	2409	6.58	4.57–8.89	**0.0036**	98.2	**0**
	mRNA vs. vector	33	21,124	2468	6.60	4.34–9.19	0.0788	97.9	**0**
	Vector vs. inactivated	17	13,538	651	1.37	0.11–3.51	0.5969	95.8	** < 0.0001**
Disease category	Rheumatologic and musculoskeletal	34	43,894	3020	7.25	5.2–9.58	** < 0.0001**	96.1	**0**
	Gastroenterological	6	9832	1395	7.86	1.61–18.11		99.7	
	Dermatological	6	2386	138	4.81	1.29–9.70		89.7	
	Neurological	16	12,212	473	2.62	1.49–4.04		95.2	
	Nephrological	2	93	27	26.66	8.16–50.59		82.1	
	Hematologic	8	1186	150	14.12	3.77–28.39		95.0	

Statistically significant values (*p* < 0.05) are in bold

#### By disease category

The sample sizes of the included studies in the present systematic review and meta-analysis were heterogenous as they were as follows: rheumatic and musculoskeletal, gastroenterologic, dermatologic, neurologic, nephrologic, and hematologic disorders. To deal with the existing heterogeneity due to the disease category of the participants, we aimed to perform a subgroup meta-analysis based on their disease types. Fig. 2c and Table 2 show the proportion of relapse, flare, or exacerbation in each disease category, along with the number of observations. As illustrated, nephrologic disorders had the highest relapse, flare, or exacerbation prevalence. Thereafter, hematologic, gastroenterologic, and rheumatic disorders showed 14.12%, 7.86%, and 7.25% relapse, flare, or exacerbation, respectively. Moreover, dermatologic and neurologic disorders exhibited to have the lowest crude prevalence of relapse, flare, or exacerbation at 4.81% and 2.62%, respectively. The prevalence of relapse, flare, or exacerbation was statistically significantly different across the six disease categories overall, as shown by a *p*-value of < 0.0001. Table 2 shows complete statistical indices for this meta-analysis.

#### Rheumatologic and musculoskeletal diseases by vaccine category

Thirty-nine observations concluded from 22 studies [24, 27–29, 31, 32, 35, 43, 44, 46, 59, 60, 63, 65, 70, 77, 78, 81, 83, 90–92] were eligible to enter the subgroup meta-analysis of vaccine category among patients with rheumatologic and musculoskeletal disorders. Patients administered with mRNA vaccines showed a higher prevalence of relapse, flare, or exacerbation at 8.78% (95% CI 6.22%; 11.72%, test of heterogeneity: *I*^2^ = 92.1%), and vector-based vaccines demonstrated to have the lowest rates of relapse, flare, or exacerbation as 1.59% (95% CI 0%; 6.09%, test of heterogeneity: *I*^2^ = 95.2%). Additionally, administering vaccines on an inactive platform was shown to lead to a prevalence of 4.51% (95% CI 1.13%; 9.78%, test of heterogeneity: *I*^2^ = 94.7%) (Fig. 4b; Table 2). Testing for subgroup differences with a *p*-value of 0.0882 confirmed that the existing between-group difference was not statistically significant. Furthermore, the funnel plot was symmetric, showing no publication bias (Additional file 1: Fig. S1a).

#### Neurologic diseases by vaccine category

Thirteen observations of nine studies [19–21, 37–39, 42, 54, 76] were included in this analysis. Therefore, we conducted a subgroup meta-analysis of the vaccine category among participants with neurologic disorders. The prevalence of relapse, flare, or exacerbation in mRNA and inactive groups was as follows, respectively: 2.71% (95% CI 0.89%; 5.32%, test of heterogeneity: *I*^2^ = 84.7%), 0.7% (95% CI 0.44%; 0.99%, test of heterogeneity: *I*^2^ = 0.0%) (Fig. 4c; Table 2). A *p*-value of 0.0108 implies a statistically significant difference between the mRNA and inactive vaccine groups. Also, the funnel plot was symmetric, indicating no publication bias (Additional file 1: Fig. S1b).

### Quality assessment of included studies

Quality assessment of the included studies is presented in Additional file 1: Table S1. The majority of the studies (*n* = 65) were of good quality and 9 had fair quality.

## Discussion

Our findings confirm the minimal risk (6.28%) of relapse/flare/exacerbation in AI-IMD patients after vaccination against COVID-19. This risk was minimal in patients with neurologic or dermatologic autoimmune diseases or who were vaccinated with vector-based vaccines.

Although there is a risk of relapse/flare/exacerbation after COVID-19 vaccination, several studies have shown higher rates of relapse/flare of underlying AI-IMD after COVID-19 [93, 94]. The risk of post-COVID-19 flare in patients with IBD and Takayasu arteritis was 9.8% and 28.5%, respectively. Besides, the risk of flares after COVID-19 and vaccination in patients with MS was 12.8% and 7.7%, respectively, confirming the lower risk of flare after vaccination compared to COVID-19. Of note, COVID-19-related morbidity and mortality are significantly higher in unvaccinated AI-IMD patients [95, 96]. Putting all together, vaccination against SARS-CoV-2 in AI-IMD patients not only minimizes post-COVID-19 morbidity and mortality but also has a lower risk of flare compared to infection.

The impact of COVID-19 on the immune system is significant, highlighting the development of autoantibodies in infected individuals. Notably, patients with COVID-19 have been reported to develop antinuclear antibodies (ANA) with a "nucleolar" immunofluorescence pattern, a recognized marker of scleroderma with interstitial lung disease. This association is particularly observed in individuals with more severe pulmonary conditions [97, 98]. Additionally, the development of other autoantibodies, such as anti-platelet factor 4 (anti-PF4), is related to COVID-associated immune thrombocytopenia [99]. The exploration of these autoantibodies contributes to a better understanding of the immunological dysregulation associated with COVID-19.

All-cause costs at 90 days after severe SLE flare is reported to be $27,468 in the United States in 2021 [100]. Besides the complications the patients will experience, the vaccination will decrease the burden on the healthcare system by minimizing both SARS-CoV-2 infection and disease relapse-related hospitalization and diagnostic and therapeutic costs. Hence, international vaccination protocols should recommend booster vaccines for this vulnerable population.

Although all vaccine types showed a low risk of flare/relapse/exacerbation in AI-IMD patients, patients who received vector-based vaccines less experienced flare/relapse/exacerbation. The mechanism of immunity induction is different in mRNA and vector-based vaccines, especially in AI-IMD patients [5]. Induced IgG and neutralizing antibodies are more pronounced after mRNA priming, whereas cellular immunity (CD4 and CD8 T cell levels) were higher after vector priming [101]. This more prominent humoral response after mRNA vaccination might be the main reason for the higher relapse rate following this vaccine type.

Our findings support the continued vaccination in AI-IMD patients and provide safety information for SARS-CoV-2 vaccines. We believe that the benefits of vaccination greatly outweigh the risks and are vital in controlling the pandemic. We recommend physicians strictly follow the patients with AI-IMD after vaccination to ensure timely diagnosis of potential flare/relapse to maximize the patient's outcome. In addition, the scarcity of data in some groups such as nephrology diseases might lead to statistically significant results; though its clinical significance needs more robust evidence. Of note, the booster dose administration in patients who experienced relapse/flare after any SARS-CoV-2 vaccine dose should be investigated more. Lastly, we excluded articles not written in English and did not search grey literature reducing the analysis efficiency.

### Conclusion

In conclusion, the risk of flare/relapse/exacerbation in AI-IMD patients is found to be minimal. Vaccination against COVID-19 is recommended in this population, especially with vector-based vaccines.

### Supplementary Information

**Additional file 1: Table S1**. Quality assessment using NIH tool. **Figure S1. **Funnel plot representing no publication bias in subgroup of patients with rheumatologic and musculoskeletal (A) and neurologic diseases (B).

## Data Availability

The authors stated that all information provided in this article could be shared.

## References

[1] Mohammed AH, Blebil A, Dujaili J, Rasool-Hassan BA (2020). The risk and impact of COVID-19 pandemic on immunosuppressed patients: cancer, HIV, and solid organ transplant recipients. AIDS Rev.

[2] Thanou A, Jupe E, Purushothaman M, Niewold TB, Munroe ME (2021). Clinical disease activity and flare in SLE: current concepts and novel biomarkers. J Autoimmun.

[3] Barzegar M, Vaheb S, Mirmosayyeb O, Afshari-Safavi A, Nehzat N, Shaygannejad V (2021). Can coronavirus disease 2019 (COVID-19) trigger exacerbation of multiple sclerosis? A retrospective study. Mult Scler Relat Disord.

[4] Moghadas SM, Vilches TN, Zhang K, Wells CR, Shoukat A, Singer BH (2021). The impact of vaccination on coronavirus disease 2019 (COVID-19) outbreaks in the United States. Clin Infect Dis.

[5] Mehrabi Nejad M-M, Shobeiri P, Dehghanbanadaki H, Tabary M, Aryannejad A, Haji Ghadery A (2022). Seroconversion following the first, second, and third dose of SARS-CoV-2 vaccines in immunocompromised population: a systematic review and meta-analysis. Virol J.

[6] Zhang E, Gupta A, Al-Ani A, Macrae FA, Leong RW, Christensen B (2022). Misconceptions drive COVID-19 vaccine hesistancy in individuals with inflammatory bowel disease. Can J Gastroenterol Hepatol.

[7] Sim JJL, Lim CC (2022). Influenza vaccination in systemic lupus erythematosus: efficacy, effectiveness, safety, utilization, and barriers. Am J Med.

[8] Segal Y, Calabrò M, Kanduc D, Shoenfeld Y (2017). Human papilloma virus and lupus: the virus, the vaccine and the disease. Curr Opin Rheumatol.

[9] Crowe SR, Merrill JT, Vista ES, Dedeke AB, Thompson DM, Stewart S (2011). Influenza vaccination responses in human systemic lupus erythematosus: impact of clinical and demographic features. Arthritis Rheumat.

[10] Gebre MS, Brito LA, Tostanoski LH, Edwards DK, Carfi A, Barouch DH (2021). Novel approaches for vaccine development. Cell.

[11] Smith-Garvin JE, Koretzky GA, Jordan MS (2009). T cell activation. Annu Rev Immunol.

[12] He P, Zou Y, Hu Z (2015). Advances in aluminum hydroxide-based adjuvant research and its mechanism. Human Vacc Immunother.

[13] Fragoso YD, Gomes S, Gonçalves MVM, Junior EM, de Oliveira BES, Rocha CF (2022). New relapse of multiple sclerosis and neuromyelitis optica as a potential adverse event of AstraZeneca AZD1222 vaccination for COVID-19. Mult Scler Relat Disord.

[14] Wong HS, Park K, Gola A, Baptista AP, Miller CH, Deep D (2021). A local regulatory T cell feedback circuit maintains immune homeostasis by pruning self-activated T cells. Cell.

[15] Polack FP, Thomas SJ, Kitchin N, Absalon J, Gurtman A, Lockhart S (2020). Safety and efficacy of the BNT162b2 mRNA Covid-19 vaccine. N Engl J Med.

[16] Baden LR, El Sahly HM, Essink B, Kotloff K, Frey S, Novak R (2020). Efficacy and safety of the mRNA-1273 SARS-CoV-2 vaccine. N Engl J Med.

[17] Mehrabi Nejad M-M, Moosaie F, Dehghanbanadaki H, Haji Ghadery A, Shabani M, Tabary M (2022). Immunogenicity of COVID-19 mRNA vaccines in immunocompromised patients: a systematic review and meta-analysis. Eur J Med Res.

[18] Study Quality Assessment Tools. Available from: https://www.nhlbi.nih.gov/health-topics/study-quality-assessment-tools.

[19] Achiron A, Dolev M, Menascu S, Zohar DN, Dreyer-Alster S, Miron S (2021). COVID-19 vaccination in patients with multiple sclerosis: what we have learnt by February 2021. Mult Scler J.

[20] Ali Sahraian M, Ghadiri F, Azimi A, Naser MA (2021). Adverse events reported by Iranian patients with multiple sclerosis after the first dose of Sinopharm BBIBP-CorV. Vaccine.

[21] Alonso R, Chertcoff A, Leguizamón FDV, Galleguillos Goiry L, Eizaguirre MB, Rodríguez R (2021). Evaluation of short-term safety of COVID-19 vaccines in patients with multiple sclerosis from Latin America. Mult Scler J Exp Transl Clin.

[22] Alroughani R, Al-Hashel J, Abokalawa F, Almojel M, Ahmed SF (2022). COVID-19 vaccination in people with multiple sclerosis. The Kuwait experience. Mult Scler Relat Disord.

[23] Apaydin H, Erden A, Guven SC, Armagan B, Konak HE, Polat B (2022). Effects of anti-SARS-CoV-2 vaccination on safety and disease exacerbation in patients with Behcet syndrome in a monocentric cohort. Int J Rheum Dis.

[24] Assawasaksakul T, Lertussavavivat T, Sathitratanacheewin S, Oudomying N, Vichaiwattana P, Wanlapakorn N (2022). Comparison of immunogenicity and safety of inactivated, adenovirus-vectored and heterologous adenovirus-vectored/mRNA vaccines in patients with systemic lupus erythematosus and rheumatoid arthritis: a prospective cohort study. Vaccines.

[25] Assawasaksakul T, Sathitratanacheewin S, Vichaiwattana P, Wanlapakorn N, Poovorawan Y, Avihingsanon Y (2022). Immunogenicity of the third and fourth BNT162b2 mRNA COVID-19 boosters and factors associated with immune response in patients with SLE and rheumatoid arthritis. Lupus Sci Med.

[26] Baars AE, Kuitwaard K, de Koning LC, Luijten LWG, Kok WM, Eftimov F (2022). SARS-CoV-2 vaccination safety in Guillain-Barré syndrome, chronic inflammatory demyelinating polyneuropathy, and multifocal motor neuropath. Neurology.

[27] Barbhaiya M, Levine JM, Bykerk VP, Jannat-Khah D, Mandl LA (2021). Systemic rheumatic disease flares after SARS-CoV-2 vaccination among rheumatology outpatients in New York City. Ann Rheum Dis.

[28] Barbhaiya M, Levine JM, Siegel CH, Bykerk VP, Jannat-Khah D, Mandl LA (2022). Adverse events and disease flares after SARS-CoV-2 vaccination in patients with systemic lupus erythematosus. Clin Rheumatol.

[29] Bixio R, Bertelle D, Masia M, Pistillo F, Carletto A, Rossini M (2021). Incidence of disease flare after BNT162b2 coronavirus disease 2019 vaccination in patients with rheumatoid arthritis in remission. ACR Open Rheumatol.

[30] Brunn JA, Dunietz GL, Romeo AR, Braley TJ (2022). SARS-CoV-2 infection and vaccination outcomes in multiple sclerosis. Neurol Clin Pract.

[31] Cherian S, Paul A, Ahmed S, Alias B, Manoj M, Santhosh AK (2021). Safety of the ChAdOx1 nCoV-19 and the BBV152 vaccines in 724 patients with rheumatic diseases: a post-vaccination cross-sectional survey. Rheumatol Int.

[32] Connolly CM, Ruddy JA, Boyarsky BJ, Barbur I, Werbel WA, Geetha D (2022). Disease flare and reactogenicity in patients with rheumatic and musculoskeletal diseases following two-dose SARS-CoV-2 messenger RNA vaccination. Arthritis Rheumatol.

[33] Conticini E, d'Alessandro M, Grazzini S, Fornaro M, Sabella D, Lopalco G (2022). Relapses of idiopathic inflammatory myopathies after vaccination against COVID-19: a real-life multicenter Italian study. Intern Emerg Med.

[34] Crickx E, Moulis G, Ebbo M, Terriou L, Briantais A, Languille L (2021). Safety of anti-SARS-CoV-2 vaccination for patients with immune thrombocytopenia. Br J Haematol.

[35] Delvino P, Cassione EB, Biglia A, Quadrelli VS, Bartoletti A, Montecucco C (2022). Safety of BNT162b2 mRNA COVID-19 vaccine in a cohort of elderly, immunocompromised patients with systemic vasculitis. Clin Exp Rheumatol.

[36] Dijk WEMV, Schutgens REG (2022). Relapse of immune thrombocytopenia after COVID-19 vaccination. Eur J Haematol.

[37] Dinoto A, Gastaldi M, Iorio R, Marini S, Damato V, Farina A (2022). Safety profile of SARS-CoV-2 vaccination in patients with antibody-mediated CNS disorders. Mult Scler Relat Disord.

[38] Doron A, Piura Y, Vigiser I, Kolb H, Regev K, Nesher N (2022). BNT162b2 mRNA COVID-19 vaccine three-dose safety and risk of COVID-19 in patients with myasthenia gravis during the alpha, delta, and omicron waves. J Neurol.

[39] Dreyer-Alster S, Menascu S, Mandel M, Shirbint E, Magalashvili D, Dolev M (2022). COVID-19 vaccination in patients with multiple sclerosis: safety and humoral efficacy of the third booster dose. J Neurol Sci.

[40] Elkharsawi A, Arnim UV, Schmelz R, Sander C, Stallmach A, Teich N (2022). SARS-CoV-2 vaccination does not induce relapses of patients with inflammatory bowel disease. Z Gastroenterol.

[41] Ellul P, Revés J, Abreu B, Chaparro M, Gisbert JP, Allocca M (2022). Implementation and short-term adverse events of anti-SARS-CoV-2 vaccines in inflammatory bowel disease patients: an international web-based survey. J Crohns Colitis.

[42] Etemadifar M, Abhari AP, Nouri H, Sigari AA, Daliyeh SMP, Maracy MR (2022). Self-Reported safety of the BBIBP-CorV (Sinopharm) COVID-19 vaccine among Iranian people with multiple sclerosis. Human Vacc Immunother.

[43] Fan Y, Geng Y, Wang Y, Deng X, Li G, Zhao J (2022). Safety and disease flare of autoimmune inflammatory rheumatic diseases: a large real-world survey on inactivated COVID-19 vaccines. Ann Rheum Dis.

[44] Fornaro M, Venerito V, Iannone F, Cacciapaglia F (2022). Safety profile and low risk of disease relapse after BNT162b2 mRNA SARS-CoV-2 vaccination in patients with rare rheumatic diseases. J Rheumatol.

[45] Fragoulis GE, Bournia VK, Mavrea E, Evangelatos G, Fragiadaki K, Karamanakos A (2022). COVID-19 vaccine safety and nocebo-prone associated hesitancy in patients with systemic rheumatic diseases: a cross-sectional study. Rheumatol Int.

[46] Gaur P, Agrawat H, Shukla A (2021). COVID-19 vaccine hesitancy in patients with systemic autoimmune rheumatic disease: an interview-based survey. Rheumatol Int.

[47] Geisen UM, Berner DK, Tran F, Sümbül M, Vullriede L, Ciripoi M (2021). Immunogenicity and safety of anti-SARS-CoV-2 mRNA vaccines in patients with chronic inflammatory conditions and immunosuppressive therapy in a monocentric cohort. Ann Rheum Dis.

[48] Gerosa M, Schioppo T, Argolini LM, Sciascia S, Ramirez GA, Moroni G (2022). The impact of anti-SARS-CoV-2 vaccine in patients with systemic lupus erythematosus: a multicentre cohort study. Vaccines (Basel).

[49] Giuffrida G, Markovic U, Condorelli A, Calagna M, Grasso S, Duminuco A (2022). Relapse of immune-mediated thrombotic thrombocytopenic purpura following mRNA COVID-19 vaccination: a prospective cohort study. Haematologica.

[50] Huang YW, Tsai TF (2021). Exacerbation of psoriasis following COVID-19 vaccination: report from a single center. Front Med (Lausanne).

[51] Ishizuchi K, Takizawa T, Sekiguchi K, Motegi H, Oyama M, Nakahara J (2022). Flare of myasthenia gravis induced by COVID-19 vaccines. J Neurol Sci.

[52] Isnardi CA, Schneeberger EE, Kreimer JL, Luna PC, Echeverria C, Roberts K (2022). An Argentinean cohort of patients with rheumatic and immune-mediated diseases vaccinated for SARS-CoV-2: the SAR-CoVAC Registry-protocol and preliminary data. Clin Rheumatol.

[53] Izmirly PM, Kim MY, Samanovic M, Fernandez-Ruiz R, Ohana S, Deonaraine KK (2022). Evaluation of immune response and disease status in systemic lupus erythematosus patients following SARS-CoV-2 vaccination. Arthritis Rheumatol.

[54] Kavosh A, Ashtari F, Naghavi S, Adibi I, Shaygannejad V, Karimi Z (2022). Safety of Sinopharm vaccine for people with multiple sclerosis: study of adverse reactions and disease activity. Mult Scler Relat Disord.

[55] Kianfar N, Dasdar S, Farid AS, Balighi K, Mahmoudi H, Daneshpazhooh M (2022). Exacerbation of autoimmune bullous diseases after severe acute respiratory syndrome coronavirus 2 vaccination: is there any association?. Front Med.

[56] Kulikowska J, Kapica-Topczewska K, Czarnowska A, Tarasiuk J, Kochanowicz J, Brola W (2022). Safety of vaccines against SARS-CoV-2 among patients with multiple sclerosis treated with disease modifying therapies. Eur J Neurol.

[57] Larsen ES, Nilsson AC, Möller S, Voss AB, Johansen IS (2022). Immunogenicity and risk of disease flare after a three-dose regimen with SARS-CoV-2 vaccination in patients with systemic lupus erythematosus: results from the prospective cohort study COVAC-SLE. Clin Exp Rheumatol.

[58] Lev-Tzion R, Focht G, Lujan R, Mendelovici A, Friss C, Greenfeld S (2022). COVID-19 vaccine is effective in inflammatory bowel disease patients and is not associated with disease exacerbation. Clin Gastroenterol Hepatol.

[59] Li H, Dalbeth N, Wallace ZS, Sparks JA, Li X, Zeng C (2022). Risk of gout flares after COVID-19 vaccination: a case-crossover study. Semin Arthritis Rheumat.

[60] Li X, Tong X, Wong ICK, Peng K, Chui CSL, Lai FTT (2022). Lack of inflammatory bowel disease flare-up following two-dose BNT162b2 vaccine: a population-based cohort study. Gut.

[61] Li X, Tong X, Yeung WWY, Kuan P, Yum SHH, Chui CSL (2022). Two-dose COVID-19 vaccination and possible arthritis flare among patients with rheumatoid arthritis in Hong Kong. Ann Rheum Dis.

[62] Machado PM, Lawson-Tovey S, Strangfeld A, Mateus EF, Hyrich KL, Gossec L (2022). Safety of vaccination against SARS-CoV-2 in people with rheumatic and musculoskeletal diseases: results from the EULAR Coronavirus Vaccine (COVAX) physician-reported registry. Ann Rheum Dis.

[63] Mohanasundaram K, Santhanam S, Natarajan R, Murugesan H, Nambi T, Chilikuri B (2022). Covid-19 vaccination in autoimmune rheumatic diseases: a multi-center survey from southern India. Int J Rheum Dis.

[64] Mok CC, Chan KL, Tse SM (2022). Hesitancy for SARS-CoV-2 vaccines and post-vaccination flares in patients with systemic lupus erythematosus. Vaccine.

[65] Mormile I, Della Casa F, Petraroli A, Furno A, Granata F, Portella G (2022). Immunogenicity and safety of mRNA Anti-SARS-CoV-2 vaccines in patients with systemic lupus erythematosus. Vaccines (Basel).

[66] Musetti C, Fornara L, Cantaluppi V (2022). Clinical evaluation of immunological and clinical recurrence of immune-mediated nephropathies after SARS-COV-2 vaccine. Nephrol Dial Transplant.

[67] Musumeci ML, Caruso G, Trecarichi AC, Micali G (2022). Safety of SARS-CoV-2 vaccines in psoriatic patients treated with biologics: a real life experience. Dermatol Ther.

[68] Nakafero G, Grainge MJ, Card T, Mallen CD, Nguyen Van-Tam JS, Williams HC (2022). Is vaccination against Covid-19 associated with autoimmune rheumatic disease flare? A self-controlled case series analysis. Rheumatology (Oxford).

[69] Nakagawa N, Maruyama S, Kashihara N, Narita I, Isaka Y (2022). New-onset and relapse of nephrotic syndrome following COVID-19 vaccination: a questionnaire survey in Japan. Clin Exp Nephrol.

[70] Ozdede A, Guner S, Ozcifci G, Yurttas B, Dincer ZT, Atli Z (2022). Safety of SARS-CoV-2 vaccination in patients with Behcet's syndrome and familial Mediterranean fever: a cross-sectional comparative study on the effects of M-RNA based and inactivated vaccine. Rheumatol Int.

[71] Ozgen Z, Aksoy H, Cakici OA, Aksu AEK, Erdem O, Polat AK (2022). COVID-19 severity and SARS-Cov-2 vaccine safety in pemphigus patients. Dermatol Ther.

[72] Pan CX, Goldman N, Kim DY, Rowley R, Schaefer M, LaChance AH (2022). Disease flare in patients with dermatomyositis following COVID-19 vaccination. J Am Acad Dermatol.

[73] Pinte L, Negoi F, Ionescu GD, Caraiola S, Balaban DV, Badea C (2021). COVID-19 vaccine does not increase the risk of disease flare-ups among patients with autoimmune and immune-mediated diseases. J Pers Med.

[74] Rider LG, Parks CG, Wilkerson J, Schiffenbauer AI, Kwok RK, Farhadi PN (2022). Baseline factors associated with self-reported disease flares following COVID-19 vaccination among adults with systemic rheumatic disease: results from the COVID-19 global rheumatology alliance vaccine survey. Rheumatology.

[75] Sattui SE, Liew JW, Kennedy K, Sirotich E, Putman M, Moni TT (2021). Early experience of COVID-19 vaccination in adults with systemic rheumatic diseases: results from the COVID-19 Global Rheumatology Alliance Vaccine Survey. RMD Open.

[76] Shapiro Ben David S, Potasman I, Rahamim-Cohen D (2021). Rate of recurrent Guillain-Barré syndrome after mRNA COVID-19 vaccine BNT162b2. JAMA Neurol.

[77] Shechtman L, Lahad K, Livneh A, Grossman C, Druyan A, Giat E (2022). Safety of the BNT162b2 mRNA COVID-19 vaccine in patients with familial Mediterranean fever. Rheumatology.

[78] Spinelli FR, Favalli EG, Garufi C, Cornalba M, Colafrancesco S, Conti F (2022). Low frequency of disease flare in patients with rheumatic musculoskeletal diseases who received SARS-CoV-2 mRNA vaccine. Arthritis Res Ther.

[79] Sprow G, Afarideh M, Dan J, Feng R, Keyes E, Grinnell M (2022). Autoimmune skin disease exacerbations following COVID-19 vaccination. Front Immunol.

[80] Stastna D, Menkyova I, Drahota J, Hrnciarova T, Havrdova EK, Vachova M (2022). To be or not to be vaccinated: the risk of MS or NMOSD relapse after COVID-19 vaccination and infection. Mult Scler Relat Disord.

[81] Tang Q, Li F, Tian J, Kang J, He JS (2023). Attitudes towards and safety of the SARS-CoV-2 inactivated vaccines in 188 patients with systemic lupus erythematosus: a post-vaccination cross-sectional survey. Clin Exp Med.

[82] Trunk AD, Shewan S, Lee CJ, Couriel DR (2021). Chronic graft-versus-host disease (CGVHD) exacerbation after SARS-CoV-2 COVID vaccination. Blood.

[83] Tzioufas AG, Bakasis AD, Goules AV, Bitzogli K, Cinoku II, Chatzis LG (2021). A prospective multicenter study assessing humoral immunogenicity and safety of the mRNA SARS-CoV-2 vaccines in Greek patients with systemic autoimmune and autoinflammatory rheumatic diseases. J Autoimmun.

[84] Urra Pincheira A, Alnajjar S, Katzberg H, Barnett C, Daniyal L, Rohan R (2022). Retrospective study on the safety of COVID-19 vaccination in myasthenia gravis. Muscle Nerve.

[85] Vacchi C, Testoni S, Visentini M, Zani R, Lauletta G, Gragnani L (2022). COVID-19 vaccination rate and safety profile in a multicentre Italian population affected by mixed cryoglobulinaemic vasculitis. Clin Exp Rheumatol.

[86] Visentini M, Gragnani L, Santini SA, Urraro T, Villa A, Monti M (2022). Flares of mixed cryoglobulinaemia vasculitis after vaccination against SARS-CoV-2. Ann Rheum Dis.

[87] Visser C, Swinkels M, van Werkhoven ED, Croles FN, Noordzij-Nooteboom HS, Eefting M (2022). COVID-19 vaccination in patients with immune thrombocytopenia. Blood Adv.

[88] Weaver KN, Zhang X, Dai X, Watkins R, Adler J, Dubinsky MC (2021). Impact of SARS-CoV-2 vaccination on inflammatory bowel disease activity and development of vaccine-related adverse events: results from PREVENT-COVID. Inflamm Bowel Dis.

[89] Woolley P, Tailor A, Shah R, Westwood JP, Scully M (2022). Real-world, single-center experience of SARS-CoV-2 vaccination in immune thrombocytopenia. J Thromb Haemostasis.

[90] Yoshida T, Tsuji H, Onishi A, Takase Y, Shirakashi M, Onizawa H (2022). Medium-term impact of the SARS-CoV-2 mRNA vaccine against disease activity in patients with systemic lupus erythematosus. Lupus Sci Med.

[91] Zavala-Flores E, Salcedo-Matienzo J, Quiroz-Alva A, Berrocal-Kasay A (2022). Side effects and flares risk after SARS-CoV-2 vaccination in patients with systemic lupus erythematosus. Clin Rheumatol.

[92] Zeng HQ, Liu HJ, Liu Z, Zhou XK, Lu XP, Yan ZB (2022). Safety and immunogenicity of inactivated COVID-19 vaccination in adult rheumatic patients in South China: a prospective study. Human Vacc Immunother.

[93] Gad AHE, Ahmed SM, Garadah MYA, Dahshan A (2022). Multiple sclerosis patients’ response to COVID-19 pandemic and vaccination in Egypt. Egypt J Neurol Psychiatry Neurosurg.

[94] Karakas A, Inel TY, Onen F, Sari I (2022). The effect of COVID-19 pandemic in a large series of patients with Takayasu arteritis. Turk J Med Sci.

[95] Mehrabi Nejad M-M, Abkhoo A, Salahshour F, Salehi M, Gity M, Komaki H (2022). Chest CT scan features to predict COVID-19 patients’ outcome and survival. Radiol Res Pract.

[96] Salahshour F, Mehrabinejad M-M, Nassiri Toosi M, Gity M, Ghanaati H, Shakiba M (2021). Clinical and chest CT features as a predictive tool for COVID-19 clinical progress: introducing a novel semi-quantitative scoring system. Eur Radiol.

[97] Muratori P, Lenzi M, Muratori L, Granito A (2021). Antinuclear antibodies in COVID 19. Clin Transl Sci.

[98] Pascolini S, Vannini A, Deleonardi G, Ciordinik M, Sensoli A, Carletti I (2021). COVID-19 and immunological dysregulation: can autoantibodies be useful?. Clin Transl Sci.

[99] Pascolini S, Granito A, Muratori L, Lenzi M, Muratori P (2021). Coronavirus disease associated immune thrombocytopenia: causation or correlation?. J Microbiol Immunol Infect.

[100] Hammond ER, Desta B, Near AM, Wang X, Jiang M (2021). Frequency, severity and costs of flares increase with disease severity in newly diagnosed systemic lupus erythematosus: a real-world cohort study, United States, 2004–2015. Lupus Sci Med.

[101] Schmidt T, Klemis V, Schub D, Schneitler S, Reichert MC, Wilkens H (2021). Cellular immunity predominates over humoral immunity after homologous and heterologous mRNA and vector-based COVID-19 vaccine regimens in solid organ transplant recipients. Am J Transplant.

