# Supplementary material for: Risk of flare or relapse in patients with immune-mediated diseases following SARS-CoV-2 vaccination: a systematic review and meta-analysis
Source: Eur J Med Res. 2024 Jan 17;29:55. doi: 10.1186/s40001-024-01639-4 (PMC10792904; doi:10.1186/s40001-024-01639-4)
Supplement: Supplementary file 1 — Additional file 1: Table S1. Quality assessment using NIH tool. Figure S1. Funnel plot representing no publication bias in subgroup of patients with rheumatologic and musculoskeletal (A) and neurologic diseases (B). [file 40001_2024_1639_MOESM1_ESM.docx]

| **Table S1: Quality assessment using NIH tool.** | | | | | |
| --- | --- | --- | --- | --- | --- |
| **First Author** | **Sum/14** | **First Author** | **Sum/14** | **First Author** | **Sum/14** |
| Achiron A | 11 | Fragoulis G | 13 | Özgen Z | 11 |
| Adája E Baars | 12 | Gaur P | 10 | Pan CX | 9 |
| Alonso R | 13 | Geisen M | 12 | Pinte L | 11 |
| Alroughani R | 9 | Gerosa M | 13 | Rider L | 12 |
| Apaydin H | 13 | Giuffrida | 12 | Sahraian MA | 12 |
| Assawasaksaku T | 12 | Huang YW | 12 | Sattui S | 14 |
| Assawasaksakul T | 12 | Ishizuchi K | 11 | Shapiro Ben David S | 11 |
| Barbhaiya M | 11 | Isnardi C | 12 | Shechtman L | 13 |
| Barbhaiya M | 11 | Izmirly P | 12 | Spinelli FR | 12 |
| Bixio R | 11 | Kavosh A | 12 | Sprow G | 12 |
| Brunn JA | 10 | Kianfar N | 13 | Stastna D | 11 |
| Cherian S | 12 | Larsen E | 13 | Tang Q | 13 |
| Connolly CM | 13 | Lev-Tzion R | 11 | Trunk AD | 10 |
| Conticini C | 12 | Li H | 12 | Tzioufas AG | 13 |
| Crickx E | 11 | Li X | 12 | Urra Pincheira A | 11 |
| Czarnowska A | 12 | Li X | 11 | Vacchi C | 11 |
| Delvino F | 12 | Machado PM | 14 | van Dijk W | 13 |
| Dinoto A | 12 | Mohanasundaram K | 13 | Visentini M | 9 |
| Doron A | 14 | Mok CC | 14 | Visser C | 12 |
| Dreyer-Alster S | 12 | Mormile I | 13 | Weaver KN | 11 |
| Elkharsawi A | 10 | Musetti C | 13 | Woolley P | 10 |
| Ellul p | 13 | Musumeci M | 12 | Yoshida Y | 13 |
| Etemadifar M | 12 | Nakafero G | 11 | Zavala-Flores E | 10 |
| Fan Y | 11 | Nakagawa n | 12 | Zeng HQ | 12 |
| Fornaro M | 12 | Ozdede | 13 |  |  |


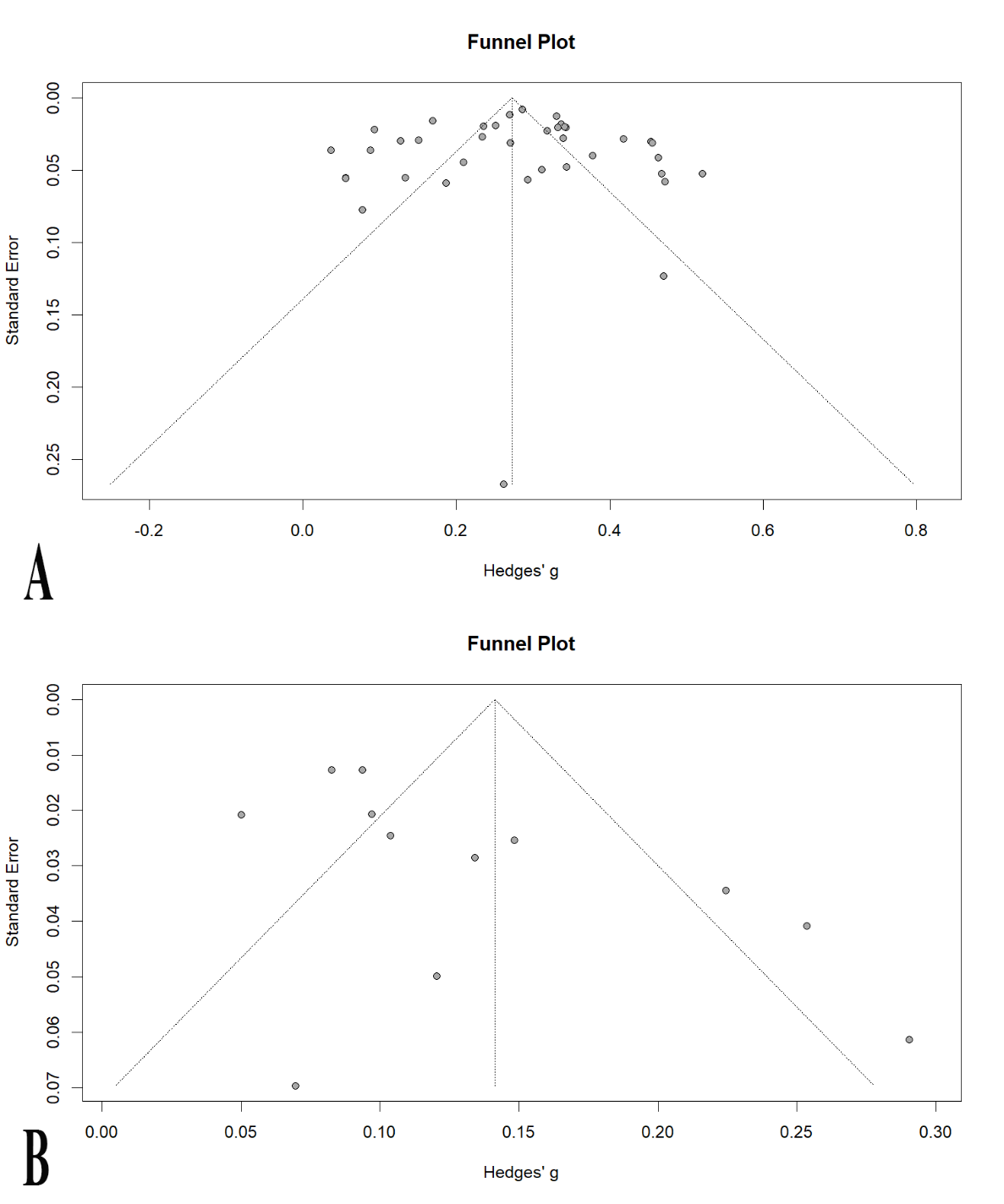


**Figure S1:** Funnel plot representing no publication bias in subgroup of patients with rheumatologic and musculoskeletal (A) and neurologic diseases (B).
